# The Spatiotemporal Pattern of Src Activation at Lipid Rafts Revealed by Diffusion-Corrected FRET Imaging

**DOI:** 10.1371/journal.pcbi.1000127

**Published:** 2008-07-25

**Authors:** Shaoying Lu, Mingxing Ouyang, Jihye Seong, Jin Zhang, Shu Chien, Yingxiao Wang

**Affiliations:** 1 Department of Bioengineering, University of Illinois at Urbana-Champaign, Urbana, Illinois, United States of America; 2 Neuroscience Program, University of Illinois at Urbana-Champaign, Urbana, Illinois, United States of America; 3 Beckman Institute for Advanced Science and Technology, Department of Molecular and Integrative Physiology and Center of Biophysics and Computational Biology, University of Illinois at Urbana-Champaign, Urbana, Illinois, United States of America; 4 Department of Pharmacology and Molecular Sciences, Johns Hopkins University, Baltimore, Maryland, United States of America; 5 Solomon H. Snyder Department of Neuroscience and Department of Oncology, Johns Hopkins University, Baltimore, Maryland, United States of America; 6 Department of Bioengineering, University of California at San Diego, San Diego, California, United States of America; 7 Department of Medicine, University of California at San Diego, San Diego, California, United States of America; Pennsylvania State University, United States of America

## Abstract

Genetically encoded biosensors based on fluorescence resonance energy transfer (FRET) have been widely applied to visualize the molecular activity in live cells with high spatiotemporal resolution. However, the rapid diffusion of biosensor proteins hinders a precise reconstruction of the actual molecular activation map. Based on fluorescence recovery after photobleaching (FRAP) experiments, we have developed a finite element (FE) method to analyze, simulate, and subtract the diffusion effect of mobile biosensors. This method has been applied to analyze the mobility of Src FRET biosensors engineered to reside at different subcompartments in live cells. The results indicate that the Src biosensor located in the cytoplasm moves 4–8 folds faster (0.93±0.06 µm^2^/sec) than those anchored on different compartments in plasma membrane (at lipid raft: 0.11±0.01 µm^2^/sec and outside: 0.18±0.02 µm^2^/sec). The mobility of biosensor at lipid rafts is slower than that outside of lipid rafts and is dominated by two-dimensional diffusion. When this diffusion effect was subtracted from the FRET ratio images, high Src activity at lipid rafts was observed at clustered regions proximal to the cell periphery, which remained relatively stationary upon epidermal growth factor (EGF) stimulation. This result suggests that EGF induced a Src activation at lipid rafts with well-coordinated spatiotemporal patterns. Our FE-based method also provides an integrated platform of image analysis for studying molecular mobility and reconstructing the spatiotemporal activation maps of signaling molecules in live cells.

## Author Summary

Fluorescence biosensors have been widely used to report the spatial and temporal activity of target molecules in live cells. However, biosensors can move independently of the target molecule and carry its signal to other subcellular locations. Therefore, the observed images appear to be the combination of the target molecular activity and the artifacts introduced by the movement of the biosensors (mainly due to diffusion). The intriguing question is how to estimate and exclude the movement effect of biosensors from the observed fluorescent images and to reconstruct the real activity map of the target molecules. The Src molecule plays important roles in cell adhesion, migration, and cancer invasion. In this paper, we developed a novel computational method to analyze and simulate the movement of the Src biosensor, which was then subtracted from the original fluorescent images. With this computational method, we observed discrete clusters of high Src activity at relatively stationary locations on the plasma membrane. Therefore, our results highlight the coordination of molecular activities in space and time. In addition to Src, our computational method can be used to reconstruct the activity map of other signaling molecules.

## Introduction

Src is a protein tyrosine kinase which plays crucial roles in cell adhesion, migration and cancer invasion [1]. In fact, epidermal growth factor (EGF) and its receptor EGFR has been well documented to couple with Src kinase to regulate cancer progression [2]. Before stimulation, Src is localized at microtubule-associated endosomes around the nucleus [3]–[7]. The SH3 and SH2 domains of Src kinase are coupled together by intramolecular interaction, and the catalytic kinase domain of Src is masked by the interaction with C-terminal tail, thus preventing its action on substrate molecules [8]. Upon EGF stimulation, Src can translocate to focal adhesion sites and associate with actin filaments at cell periphery [4], [5], [9]–[12], possibly through the Src N-terminal tail and SH3 domain, but not the catalytic domain [3],[10],[13]. Recent evidence indicates that EGF can enhance the Src localization and activation at lipid rafts to regulate cancer development [14]–[16]. However, the existence of the extremely small and dynamic lipid rafts, and the mechanism on how these lipid rafts function as docking sites to coordinate signaling molecules, remain controversial [17],[18]. It is also not clear how EGF activates Src spatially and temporally at lipid rafts to impact on cellular functions.

Genetically encoded biosensors based on fluorescence resonance energy transfer (FRET) are powerful tools for live cell imaging [19],[20]. A variety of such biosensors utilizing cyan fluorescence protein (CFP) and yellow fluorescence protein (YFP) have been developed to visualize the activities of important kinases in live cells, including epithelial growth factor receptor (EGFR), Abl [21], protein kinase A [22], protein kinase B [23], protein kinase C [24], and insulin receptor [25]. We have also developed a genetically-encoded FRET biosensor for monitoring Src activity in live cells [21],[26]. The investigations based on these biosensors have provided invaluable information about the spatiotemporal activation pattern of the molecules studied [27],[28]. However, the observed FRET signal reported by these biosensors at any given spot represents the combined effect of two main factors: (1) the local kinase activity acting on biosensors and (2) the signal of activated biosensors moving in the cell among locations. The movement of these biosensors is not dependent on the motion of the targeting enzymes or their endogenous substrate molecules. Hence, the rapid motion of the biosensors can artificially dissipate the cumulative signals engendered by the in situ enzymatic activity. Therefore, it is essential to identify and subtract the effect of biosensor motility from the apparent FRET signals to allow an accurate reconstruction of the spatiotemporal activation map of the targeting kinase.

The fluorescence recovery after photobleaching (FRAP) analysis has been widely used to estimate the apparent diffusion coefficients and characterize the motion of fluorescent molecules in live cells [29]–[32]. In classical FRAP analysis, the fluorescence recovery curve is obtained by monitoring the average fluorescence intensity in a small region after photobleaching. Based on the recovery curve, the apparent diffusion coefficient of fluorescent molecules can be estimated by parameter fitting [29]. However, this approach has specific requirements on the cell geometry, photobleached spot, and the actual photobleaching process [29]–[31],[33]. Most recently, FRAP analysis using numerical methods, such as the computational particle method, the finite difference method, and the Monte Carlo simulation, have been developed to address these limitations [34]–[40]. Results from FRAP analysis have revealed the characteristics of transport kinetics for many important molecules [41]–[43]. Nonetheless, there is a need to apply these methods to quantify and analyze live-cell FRET images.

The finite element (FE) method is well known for its flexibility in resolving the complex geometry of tissue and cellular structures [44],[45]. It has been used to estimate the apparent diffusion constant in inhomogeneous tissues [46] and for modeling protein transport in single cells [47]. In this study, we have developed a new imaging analysis method based on FE and FRAP to evaluate the motility of different Src biosensors. The results revealed that the motility of biosensors tethered to lipid rafts is governed by 2D diffusion. After the effect of biosensor diffusion on FRET signals was subtracted from the apparent FRET images, the diffusion-corrected FRET signals revealed that, at lipid rafts, high Src activities upon EGF stimulation are concentrated at relatively stationary clusters around cell periphery. Our FE-based imaging analysis method, integrated with FRAP and FRET technologies, can also serve as a general method to study the spatiotemporal kinetics of other enzymatic activity in living cells.

## Results

### Computer Simulation and Validation

**Figure 1:**
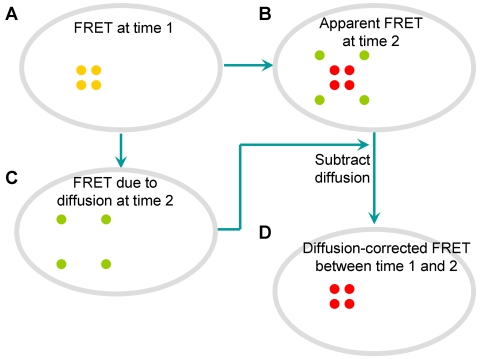
A schematic illustration on using FE analysis to compute the kinase activity map from apparent FRET video images. (A) An image showing the original location of activated FRET biosensors. (B) A possible apparent FRET signals at the next time step, where newly activated biosensors (hence actions of kinase) are mixed with those translocated from (A) due to diffusion. (C) The simulated FRET distribution map of the biosensor due to diffusion from (A). (D) The actions of kinase activity detected by subtracting (C) from (B). Yellow or red dots represent the FRET biosensors activated by the target kinase at different time steps. Green dots represent the activated biosensors translocated from other locations due to diffusion.

**Figure 2:**
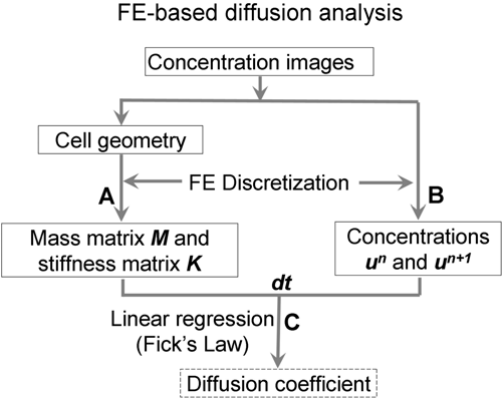
The flow chart representing the application of FE method to discretize the cell geometry and calculate diffusion coefficient using Fick's Law and linear regression based on two consecutive concentration images and the time interval (*dt*). Step (A) shows the computation of stiffness matrix *K* and the mass matrix *M* based on the FE discretization on the cell geometry. In step (B), the concentration vectors (*u^n^* and *u^n+1^*) are obtained by collecting the concentration values at the nodes of FE discretization in the images. In step (C), the least-square linear fitting is used to estimate the diffusion coefficient based on all the information from the previous steps: *M*, *K*, *u* ^n^, *u^n+1^* and *dt*.

To assess the effect of biosensor diffusion on the apparent FRET images recorded in experiments (Figure 1), we developed a FE-based method to analyze protein diffusion in FRAP experiments. Based on Fick's second law of diffusion, the change of molecular concentration in time is proportional to the second derivative of the concentration in space, i.e., the Laplacian of concentration. This can be expressed mathematically as following:
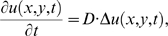
(1)where 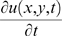 represents the time derivative of the concentration *u(x,y,t)* at a given time and location in 2D space, Δ*u*(*x,y,t*) denotes the Laplacian of *u(x,y,t)* and *D* represents the diffusion coefficient of the target molecule [33]. After Eq. (1) was discretized using the FE method, the apparent diffusion coefficient can be estimated by applying a linear regression procedure on the weighted discrete Laplacian of concentration (WDLC) and the weighted change of concentration in time (WCCT) (Figure 2, and see Materials and Methods, “Computational Simulation and Validation of the Diffusion Model”).

**Figure 3:**
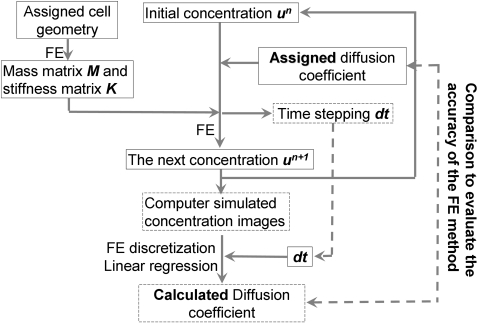
The computational procedure used to simulate FRAP experiment and calculate diffusion coefficient to evaluate the accuracy of the FE-analysis. With an assigned cell geometry, an initial concentration, and a diffusion coefficient, a series of concentration images at later time steps were generated using the finite element method and diffusion model. The simulated concentration maps were then used as the input to calculate diffusion coefficients using FE analysis and linear regression. This calculated diffusion coefficient was compared with the assigned diffusion coefficient to examine the accuracy of the method. The main output of this procedure is the simulated concentration images and the estimated diffusion coefficient as outlined in the dashed boxes. The data connected by dashed line arrows were shared between different layers.

**Figure 4:**
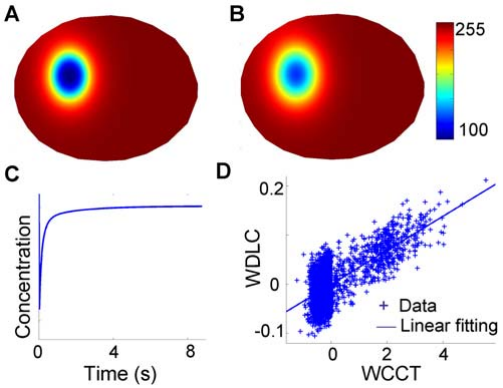
The validation of the FE-based method using computer simulation. (A) The initial concentration map of a diffusive fluorescence molecule in a single cell; (B) the concentration map at 0.0313 s produced by computer simulation with an assigned diffusion coefficient of 29 µm^2^/sec; (C) the simulated fluorescence recovery curve for 9 sec after photobleaching; (D) the scattered plot of the WDLC, −*dt*·*K*·0.5(*u^n^*+*u^n^* ^+1^), vs. the WCCT, *M*(*u^n^* ^+1^−*u^n^*), on each mesh node. Linear fitting is represented by the solid line.

The FE-based image analysis method was validated by computational modeling of the diffusion process (Figure 3). A designated cell geometry, an initial distribution of molecular concentration to mimic the fluorescence image after photobleaching, a diffusion coefficient of 29 µm^2^/sec (the diffusion coefficient of XPA-GFP which has the same size as our cytosolic Src biosensor [39]) were first assigned. A sequence of concentration maps (Figure 4A–4B) was numerically generated and saved to mimic the real procedures in FRAP experiments and used for the computation of the fluorescence recovery curve (Figure 4C). Based on these simulated FRAP images, FE analysis was used to triangulate the cell geometry and discretize the diffusion equation (Figures 3 and S1). Linear regression was then used to calculate the apparent diffusion coefficient (Figure 2) to be 30.3 µm^2^/sec, close to the assigned diffusion coefficient. Because the simulated diffusion process is governed by Fick's law, the WDLC should be linearly correlated to WCCT. The plot of WDLC vs. WCCT on each FE mesh-node verified a linear relationship between these two quantities (Figure 4D). All these results suggest that our method is accurate for modeling diffusion process. A large portion of data points in Figure 4D clustered near the zero of WCCT, suggesting that there was no significant change of the concentration at many mesh nodes distant from the photobleached spot over one time-step. Meanwhile, the noise in Figure 4D is likely due to image processing in the simulation to mimic the procedures of data processing in FRAP and FRET experiments (saving and loading image files), since the same discretization method was used for simulating concentration maps and estimating diffusion coefficient. These noises can indeed be eliminated by running the simulation without saving/loading images (data not shown).

### Image Analysis of FRAP Experiments

**Figure 5:**
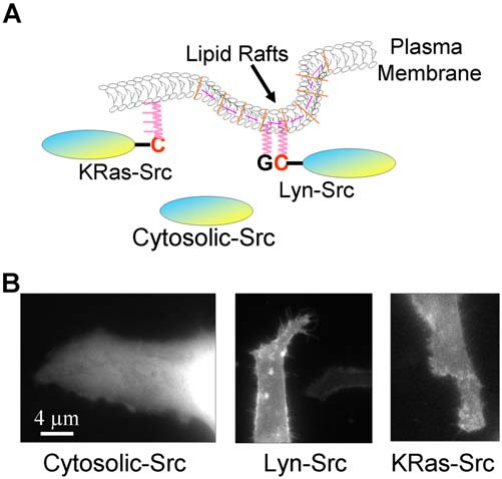
The characterization of the Src biosensors. (A) The Lyn-Src biosensor is anchored to the lipid rafts of the plasma membrane via N-terminal acylation sequences derived from the N-termini of Lyn kinase; the KRas-Src biosensor is connected to the non-lipid-rafts region through C-terminal prenylation sequences derived from KRas. Panel (B) shows the expression level of HeLa cells transfected with Cytosolic-Src, Lyn-Src, or KRas-Src biosensors, from left to right, respectively.

**Figure 6:**
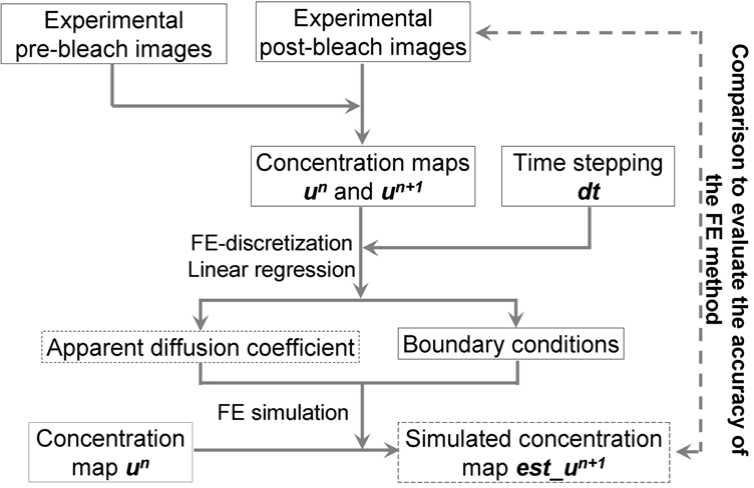
The FE analysis procedures for assessing and simulating diffusion based on experimental FRAP images are illustrated. With two consecutive experimental concentration maps and the time interval (*dt*), the apparent diffusion coefficient and boundary conditions were estimated by using our FE-based diffusion analysis. Subsequently, the diffusion coefficient and the boundary conditions were used to simulate and predict the concentration image at the next time step (*est_u^n+1^*), which was produced by allowing linear diffusion from the current image (*u^n^*). The main outputs of the described procedure are the apparent diffusion coefficient and the simulated concentration maps, as outlined in the dashed boxes.

**Figure 7:**
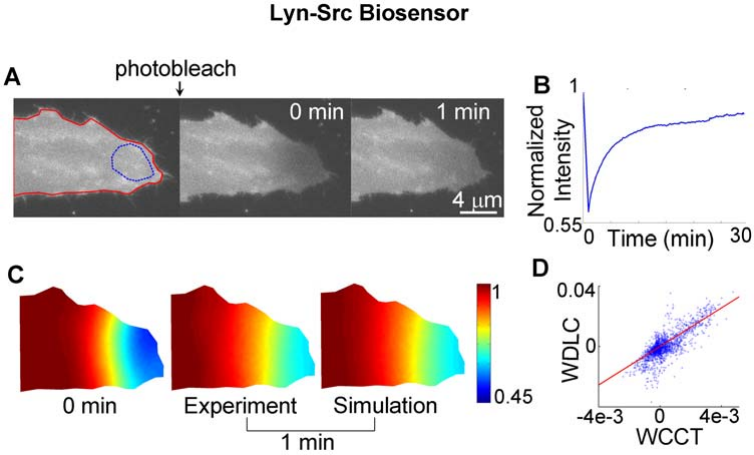
The experimental FRAP images of Lyn-Src biosensor are compared with those predicted by simulation. (A) Left: the fluorescence intensity image of a cell before photobleaching, with the red-colored outline defining the cell edge in simulation and blue-colored outline defining the region of interest monitored for fluorescence recovery. Middle and right: the fluorescence intensity images at 0 and 1 min after photobleaching, respectively. The complete time course of this FRAP experiment is shown in Movie S1. (B) The time course of fluorescence recovery in the photobleached area as marked in (A). (C) Left: the concentration map after photobleaching (0 min), computed by normalizing the fluorescence intensity with the image before photobleaching. Middle and right: the experimental and simulated concentration maps at 1 min after photobleaching. (D) The scattered plot of WDLC vs. WCCT on each mesh node, with the linear fitting indicated by the solid line.

A Src FRET biosensor was previously modified and tethered at lipid rafts in plasma membrane through a myristoylation and palmitoylation tag at the N-terminal (Lyn-Src) (see Figures S2 and 5) [26],[48]. We have further developed, analyzed, and compared two other versions of compartment-localized Src FRET biosensors as shown in Figure 5 [21],[26]. One biosensor is targeted to membrane regions outside of lipid rafts through a geranylgeranylated tag at the C-terminal (KRas-Src) [48] and the other is located in the cytoplasm and the nucleus (Cytosolic-Src). To assess their mobility, the biosensors in a small region of a live cell were photobleached. The post-bleaching images were monitored and then normalized by the pre-bleaching images to obtain concentration maps. Subsequently, the FE analysis and linear regression method were applied on the concentration maps to estimate the apparent diffusion coefficient (Figure 6). As shown in Figure 7A–7B and Movie S1, the fluorescence intensity of the Lyn-Src biosensor localized at lipid rafts recovered in ∼15 min after photobleaching, with an estimated apparent diffusion coefficient of 0.11±0.01 µm^2^/sec. To evaluate the accuracy of the diffusion model, the mobility of this Lyn-Src biosensor was simulated and compared with experimental results. The simulation-predicted concentration map of the Lyn-Src biosensor at 1 min after photobleaching precisely matches the experimental result (Figure 7C). The linear relationship between WDLC and WCCT further confirmed that the motion of the Lyn-Src biosensor is dominated by diffusion and governed by Fick's law (Figure 7D). These results suggest that our diffusion model can accurately predict the motility of biosensor tethered at lipid rafts.

**Figure 8:**
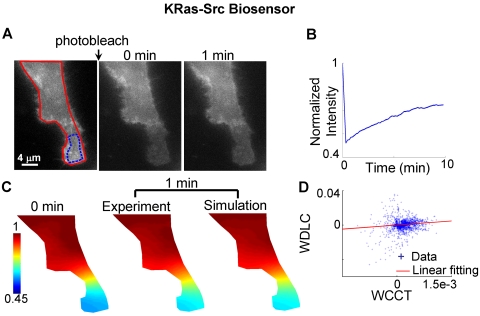
The experimental recovery images after photobleaching of Src biosensor targeted to plasma membrane outside of lipid rafts (KRas-Src biosensor) are compared with those predicted by simulation. (A) Left: the fluorescence intensity image of a cell before photobleaching, with the red-colored outline defining the cell edge in simulation and blue-colored outline defining the region of interest monitored for fluorescence recovery. Middle and right: the fluorescence intensity images at 0 and 1 min after photobleaching, respectively. (B) The time course of fluorescence recovery in the photobleached area as marked in (A). (C) Left: the concentration map after photobleaching (0 min), computed by normalizing the fluorescence intensity with the reference image before photobleaching. Middle and right: at 1 min after photobleaching, the experimental concentration map is similar to the simulation. (D) The scattered plot of the weighted Laplacian of the concentration vs. the weighted change of concentration in time on each mesh node, with the linear fitting indicated by the solid line.

**Figure 9:**
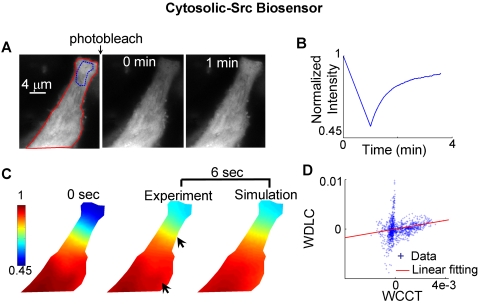
The experimental FRAP images for the cytosolic Src biosensor are compared with those predicted by simulation. (A) Left: the fluorescence intensity image of a cell before photobleaching, with the red-colored outline defining the cell edge in simulation and blue-colored outline defining the region of interest monitored for fluorescence recovery. Middle and right: the fluorescence intensity images at 0 and 1 min after photobleaching, respectively. (B) The time course of fluorescence recovery in the photobleached area as marked in (A). (C) Left: the concentration map after photobleaching (0 sec), computed by normalizing the fluorescence intensity with the image before photobleaching. Middle and right: the experimental and simulated concentration maps at 6 seconds after photobleaching, with the difference between experiment and simulate indicated by two arrows. (D) The scattered plot of the weighted Laplacian of the concentration vs. the weighted change of concentration in time on each mesh node, with the linear fitting indicated by the solid line.

Similar approaches were employed to analyze the mobility of the KRas-Src and the Cytosolic-Src biosensors (Figures 8–9). The fluorescence intensity of the Cytosolic-Src biosensor recovered in ∼4 min after photobleaching (Figure 9A–9B). The estimated apparent diffusion coefficient of the Cytosolic-Src biosensor was 0.93±0.06 µm^2^/sec, which is 4–8 folds higher than that of the membrane-bound Lyn-Src (0.11±0.01 µm^2^/sec) and KRas-Src biosensors (0.18±0.02 µm^2^/sec). These observations are consistent with previous reports that the diffusion rate of the molecules near the plasma membrane is 2- to 3-fold slower than that in cytoplasm [30],[49],[50], possibly reflecting the different nature of diffusions in 2D (membrane) and 3D (cytosolic/nucleus).

**Figure 10:**
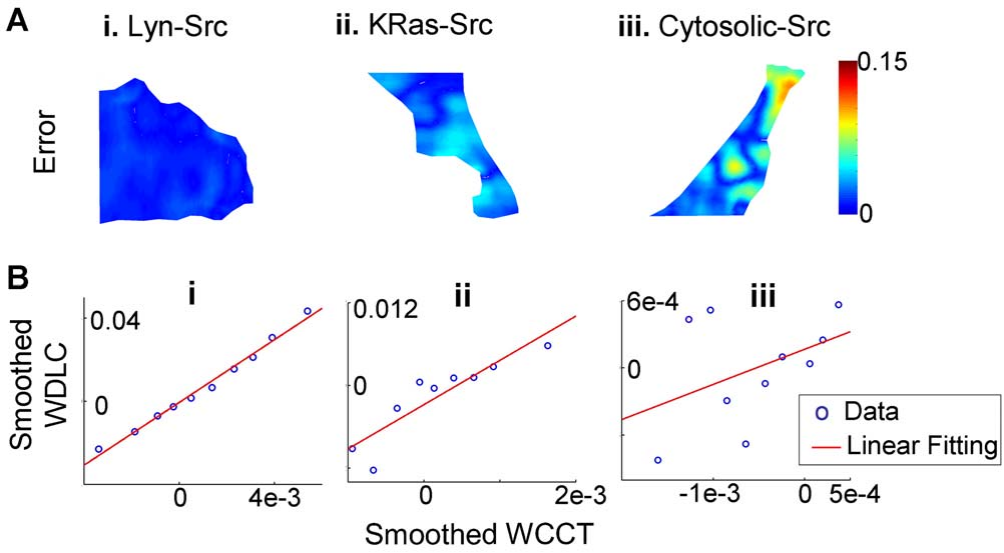
The assessment of the accuracy of the diffusion model for the Lyn-Src, KRas-Src, and Cytosolic-Src biosensors. (A) the difference (absolute value) of fluorescence intensity between simulated and experimental images for (i) Lyn-Src biosensor at 10 sec after photobleaching, (ii) KRas-Src biosensor at 10 sec after photobleaching, (iii) Cytosolic-Src biosensor at 1 sec after photobleaching because of its fast recovery rate; (B, i–iii) the scattered plots of smoothed WDLC vs. smoothed WCCT for the corresponding cells shown in (A, i–iii).

Error analysis procedures were designed to further evaluate the accuracy of the FE-based diffusion analysis for the three versions of Src biosensors (Figure 10, see Materials and Methods, “Error Analysis”). For the Lyn-Src biosensor, the model prediction matches experimental result precisely (Figure 10A_i), and the scattered linear plot of data shows high confidence with the model (Figure 10B_i). The KRas-Src biosensor also had relatively uniform distribution on plasma membrane (Figure 5B), with reasonable agreement between experimental and simulation results (Figure 10A_ii–10B_ii). It is of note that the mobility of the KRas-Src biosensor appears slightly less well predicted than for the Lyn-Src biosensor (Figure 10). On the other hand, the results for the Cytosolic-Src biosensor demonstrated an obvious disagreement between simulation and experiments (Figure 10A_iii–10B_iii), which is attributable, at least in part, to the accumulation of a large fraction of the Cytosolic-Src biosensor in the nucleus in which molecules may have significantly different mobility from that in the cytoplasm (Figure 5B).

**Figure 11:**
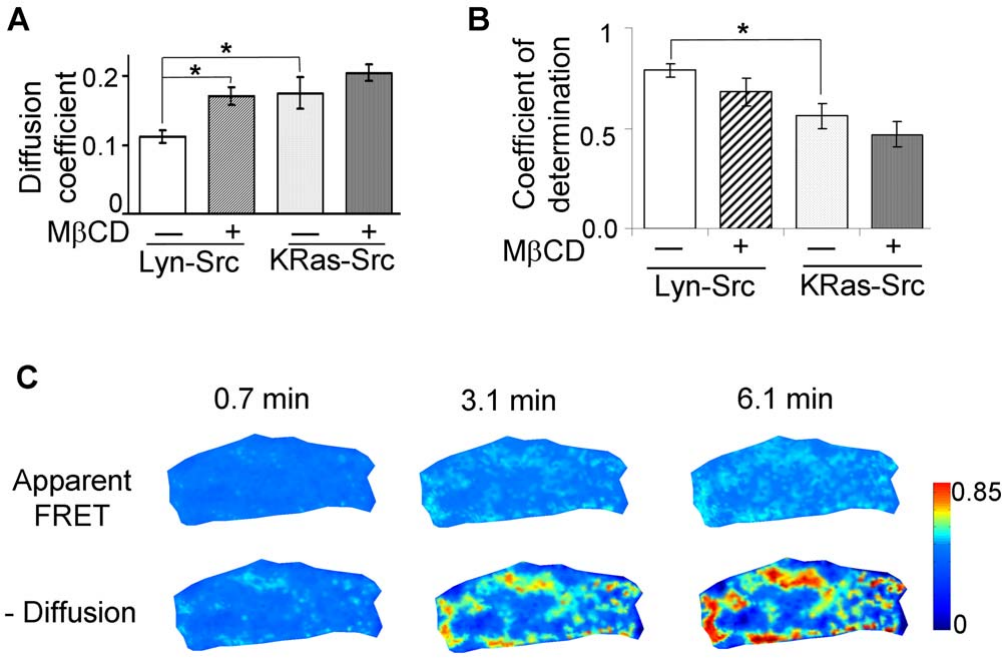
The assessment of the diffusion model accuracy and the subtraction of biosensor diffusion effects from the apparent FRET images. (A) The bar graph shows the apparent diffusion coefficients (mean±S.E.M.) of Lyn-Src and KRas-Src biosensors in control cells [0.11±0.01 µm^2^/sec (n = 43) and 0.18±0.02 µm^2^/sec (n = 17), respectively], and in cells treated with MβCD [0.17±0.01 µm^2^/sec (n = 20) and 0.20±0.01 µm^2^/sec (n = 22), respectively]. (B) The bar graph shows the coefficients of determination (mean±S.E.M.) of the diffusion models for Lyn-Src and KRas-Src biosensors in control cells [0.79±0.033 (n = 50) and 0.56±0.06 (n = 15), respectively], and in cells treated with MβCD [0.68±0.07 (n = 11) and 0.47±0.06 (n = 16), respectively]. *Asterisks* in (A) and (B) denote significant differences (*p*<0.05) between different groups as indicated. (C) The FRET signals before and after the subtraction of the effect of Lyn-Src biosensor diffusion from the apparent FRET ratio images. Top panels show the apparent FRET images and lower panels show the corresponding diffusion-subtracted FRET images of the Lyn-Src biosensor, at 0.7, 3.1, 6.1 min after EGF stimulation as indicated. The spatial-temporal dynamics of FRET signals before and after subtracting diffusion is also shown in Movie S2.

To gain more insights about the molecular dynamics and kinetics in lipid rafts, we investigated and compared the kinetics of the Lyn-Src and the KRas-Src biosensors in cells with MβCD treatment, which extracts cholesterol and disrupts lipid rafts. Without MβCD treatment, the Lyn-Src biosensor was found by FRAP analysis to move at a slower rate on the plasma membrane than the KRas-Src biosensor. Since the Lyn-Src biosensor is tethered on the lipid rafts, which are subdomains of plasma membrane rich in cholesterol [48], [50]–[53], this finding corroborates previous observations that molecules move more slowly in the cholesterol-rich than cholesterol-poor model membranes [54]. In fact, we found that the treatment with MβCD to disrupt cholesterol-associated rafts significantly increased the apparent diffusion coefficient of the Lyn-Src biosensor (from 0.11±0.01 to 0.17±0.01 µm^2^/sec), but not the KRas-Src biosensor (from 0.18±0.02 to 0.20±0.01 µm^2^/sec) (Figure 11A). This result is also consistent with earlier findings that MβCD enhances the molecular motility of HRas-tagged green fluorescence protein (GFP) tethering on lipid rafts, but not KRas-tagged GFP [50]. The large coefficient of determination (*R^2^ = 0.79±0.033*) (Figure 11B), which represents a high correlation between the experimental results and the simulated predictions by our diffusion model (see Method, “Error Analysis”), suggests that the mobility of the Lyn-Src biosensor is dominated by diffusion and hence can be accurately predicted by the diffusion model. This result is also consistent with the error analysis approach (Figure 10). The mobility of the KRas-Src biosensor (*R^2^ = 0.56±0.06*) is less well predicted by simulation, suggesting that transportation mechanisms other than 2D diffusion may also contribute to the mobility of biosensors tethered outside of lipid rafts.

### Subtracting Diffusion

The apparent FRET images of the Src biosensors represent the combinatory effects of spatiotemporal Src kinase activity and the re-distribution of mobile activated biosensors (Figure 1). Hence the apparent FRET signals may be different from the actual distribution of Src activity or its actions on endogenous substrate molecules. In fact, many prominent substrate molecules of Src kinase, e.g., p130cas and paxillin, are localized at subcellular regions with limited mobility in adherent cells [55],[56]. Recent evidence indicates that lipid rafts serve as an integrated platform for Src activation [16],[57] and the recruitment of P130cas and paxillin [58]–[60]. However, there is a lack of knowledge on the spatiotemporal pattern of Src activation at lipid rafts or its accumulative effects on the relatively immobile substrate molecules.

To reconstruct the Src activation map at lipid rafts, the contribution of biosensor diffusion was simulated and subtracted from apparent FRET signals. Error analysis has shown that the FE model of diffusion can precisely predict the movement of the Lyn-Src biosensor. Control experiments suggest that the diffusion rate of the Lyn-Src biosensor does not differ significantly with or without EGF stimulation (data not shown). Hence a diffusion coefficient of the Lyn-Src biosensor calculated before EGF stimulation can be applied to simulate the diffusion process through the entire time course of FRET experiment in the same cell. The subtraction of this simulated diffusion effect revealed discrete clusters of high Src activities at lipid rafts close to the cell edge, in contrast to the FRET images without diffusion subtraction which are relatively uniform (see Figure 11C and Movie S2) [26]. Immunostaining of the distribution of Src activity in fixed cells upon growth factor stimulation [5],[55] also showed high Src activities concentrated at cell periphery, consistent with our observations. It is of note that the locations with high Src activity at lipid rafts are relatively stationary upon EGF stimulation (Figure 11C), suggesting that active Src remains localized without significant motion upon arrival at lipid rafts.

## Discussion

The timing and localization of molecular activities are crucial for their proper functions. In this paper, we have integrated FE-based imaging analysis modeling, FRAP and FRET technologies, to reconstruct and visualize the spatiotemporal Src activity in lipid rafts upon EGF stimulation. The mobility of the Src biosensor tethered in the lipid rafts of plasma membrane was shown to be dominated by diffusion. The subtraction of this diffusion effect from FRET images has helped to reconstruct the Src activation map at lipid rafts, with high Src activity localized at stationary clusters proximal to cell edge. Given the important roles of Src and lipid rafts in mediating EGF/EGFR-regulated cancer development [2],[14], our results should shed new lights on how cells coordinate molecular activities in space and time to orchestrate pathophysiological responses upon external stimulation. The advantage of our live-cell imaging approach is further underscored by the controversial effect of non-ionic detergents used for isolating lipid rafts in traditional assays [17],[61].

Although the roles of Src in regulating downstream signaling pathways are well studied, the detailed mechanism of Src activation in response to EGF is not clearly elucidated [62]. It has been shown that growth factors can induce the translocation of Src from perinuclear regions to cell periphery through RhoB and actin cytoskeleton [5],[63]. Our results suggest that Src can be transported and activated at lipid rafts. The active Src molecules upon arrival at lipid rafts appear relatively stationary with sub-compartment localization since the activation pattern of Src biosensor showed clusters with increasing size, but little motion (Figure 11C). It has been shown that EGF can form complex with its receptor EGFR, which further binds to integrins [64]. Since integrins are anchored to immobile extracellular matrix and well documented to coordinate the localization of lipid rafts and its associated signaling molecules [65],[66], it is possible that EGF and its ligation with EGFR induce localized Src activation at lipid rafts via integrins. In fact, evidence has shown that integrin β3 can directly bind to Src through the interaction of β3 C-terminal tail and Src SH3 domain [67]. Some evidence has shown that EGFR did not colocalize with caveolae at rest state [68]. Hence it is also possible that either EGF receptor or Src is activated outside of lipid rafts and then sequestered inside lipid rafts. Further studies are warranted to elucidate the underlying mechanism for this localized and stationary Src activity at lipid rafts in response to EGF stimulation.

The motility of the Lyn-Src biosensor is dominated by diffusion, as evidenced by the close match between experimental and simulated results, and by the strong linear correlation between WDLC and WCCT (Figures 7C and 10B_i). The mobility of the KRas-Src biosensor, however, displays some nonlinear features between WDLC and WCCT (Figure 10B_ii), suggesting that it is not completely governed by 2D diffusion. Intracellular molecule mobility is influenced by molecular interaction, diffusion, and active transportation [31],[33]. Hence, molecular interaction or active transportation may contribute to the motion of KRas-Src biosensor besides diffusion. The mobility difference between KRas- and Lyn-Src biosensors may be attributable to the tight membrane-binding of the Lyn tag through deep insertion of side chains into the bilayer interior and the fluctuating membrane-binding of the KRas tag through electrostatic switches [69]. Because the membrane-tethered biosensors extend appreciably into the cytoplasm, it is also possible that some of the restricted motion at the proximity of the plasma membrane may be due to the interaction of the biosensor with the cortical actin cytoskeletal network [70]. These interactions may have particularly contributed to the motion of KRas-Src biosensor, which is not dominated by random diffusion.

Our estimated diffusion coefficient of the Cytosolic-Src biosensor is several-fold higher than those of the membrane-targeted versions. One of the possible reasons for the difference between the diffusion coefficients of the Cytosolic-Src and membrane-targeted biosensors may be the difference in the physicochemical properties of local environment, e.g. the diffusion of the Cytosolic-Src biosensors is 3D in nature whereas that of the membrane-targeted biosensors is 2D. While our diffusion model can be used to estimate the apparent diffusion coefficient and simulate diffusion process in principle, it cannot be directly applied to study the Cytosolic-Src biosensor. The low coefficient of determination (*R^2^ = 0.33±0.1*, *n = 5*) suggests that the mobility of a large portion of the Cytosolic-Src biosensors cannot be described by diffusion. This is possibly because the Cytosolic-Src biosensors reside in different sub-compartments of the cell, e.g., the nucleus vs. the cytoplasm, as shown in Figure 5B and evidenced by the results from our fluorescence loss in photobleaching (FLIP) experiments (data not shown). The movement of the Cytosolic-Src biosensor will likely be better described by a 3D and multi-compartment diffusion model.

The approach of evaluating and subtracting diffusion based on FRAP and FRET video images can also be implemented by employing other numerical methods including finite difference method, computational particle method, and Monte Carlo simulation. We decided to choose the FE-based method because it has been well-established for modeling the diffusion processes with complex geometry in 2D and 3D [44],[71]. Since the FE methods have great flexibility in resolving the complex geometry of tissue and cellular structures [44]–[47], no specific requirement on the cell geometry, the bleaching light beam, or the photobleaching process is needed in our new FRAP analysis method. Further, efficient solvers [72] and parallel implementation on distributed computers have been extensively developed for FE methods [73]. Thus, with the integration of 3D imaging techniques, e.g. confocal microscopy, our system can be conveniently extended to 3D analysis and parallel computing environment.

In summary, our FE-based method can successfully separate the effect of biosensor diffusion from the apparent FRET signals to reconstruct the diffusion-corrected spatiotemporal activation map of membrane-tethered Src kinase. The results suggest that the EGF-induced Src activation at lipid rafts has localized and stationary patterns clustered at cell periphery. This methodology can be conveniently utilized to reconstruct other molecular activation maps from those reported by indirect and diffusion-driven biosensors.

## Materials and Methods

### Cell Lines and Culture

HeLa cells (ATCC, Manassas, Virginia) were cultured in a humidified 95% air, 5% CO_2_ incubator at 37°C. The culture medium was Dulbecco's modified Eagle's medium (DMEM) supplemented with 10% fetal bovine serum, 2 mM L-glutamine, 1 unit/ml penicillin, 100 µg/ml streptomycin, and 1 mM sodium pyruvate. The cell culture reagents were obtained from Invitrogen (San Diego, CA).

### Gene Construction, DNA Plasmids, and Transient Transfection

The gene for the Cytosolic-Src biosensor was constructed as described previously [26]. In brief, this Cytosolic-Src FRET biosensor consists of a peptide derived from Src substrate molecule p130cas and a phosphotyrosine-binding domain (SH2 domain derived from c-Src), bracketed by monomeric ECFP and Citrine (an improved version of EYFP) at the N- and C-termini. The substrate peptide phosphorylated by a Src kinase can interact with the intramolecular SH2 domain, which results in a change of distance or relative orientation between ECFP and Citrine, as shown in Figure S2. The subsequent changes of FRET between ECFP and Citrine can be represented by the ECFP/Citrine emission ratio to monitor the Src activities. The membrane-targeted ECFP was constructed by PCR amplification of the monomeric ECFP with a sense primer containing the codes for N-terminal amino acids from Lyn kinase to produce a Lyn-Src biosensor [48]. For the KRas-Src biosensor, the monomeric YFP was amplified by PCR with an anti-sense primer containing the codes for C-terminal amino acids from KRas (KKKKKSKTKCVIM). For simplicity, we refer to the monomeric ECFP and Citrine by CFP and YFP respectively in text and figures. The various plasmids were transfected into HeLa cells at 80% confluence using the lipofectamine method as described by the vendor (Invitrogen, San Diego, CA).

### Microscope Imaging

For FRAP experiments, the YFP images were collected using MetaFluor 6.2 software (Molecular Devices, Sunnyvale, California) on epi-fluorescence microscopy (Zeiss, Oberkochen, Germany) with emission at 535DF25 and excitation at 495DF20 using 1% of the light source power. During imaging, the cells were kept in CO_2_-independent medium without serum (Invitrogen) at 25°C; and the objective focus was aimed near the basal side of the cell. The cells were monitored before photobleaching to confirm there was no detectable photobleaching during imaging. Photobleaching was conducted by exciting YFP at 495DF20 in a region of interest with full power of the light source for 15 sec, after which the recovery process was imaged at 1-sec and 10-sec intervals for the cytosolic and membrane-targeted Src biosensors, respectively. For FRET experiments, the HeLa cells expressing the desired Src biosensors were starved with 0.5% FBS for 36–48 hr before being subjected to EGF (50 ng/ml) stimulation. The images were collected with a 420DF20 excitation filter, a 450DRLP dichroic mirror, and two emission filters controlled by a filter changer (480DF30 for CFP and 535DF25 for FRET). The pixel-wise images of CFP/YFP emission ratio were computed to assess the FRET signals, which represent the concentration of phosphorylated Src biosensor and hence Src activity in space and time.

### Computational Simulation and Validation of the Diffusion Model

The Src biosensors were assumed to diffuse freely inside the cytoplasm or in the membrane. According to Fick's Law, the diffusion equation is given by Eq. (1) (Results, Computer Simulation and Validation).

Enclosed in the cell boundary, a triangular mesh was generated for the finite element discretization (Figure S1). A two-dimensional model was used because the thickness of a spread cell is relatively small compared to its length and width, and the photobleached region is sufficiently big (∼2 µm) such that the 3D profile of the light beam is negligible.

Using the FE method for discretizing the Laplacian operator and the Crank-Nicholson Scheme for approximating time derivative [74], Eq. (1) can be approximated by a discrete linear system (for details see Text S1, “The Formulation of the Finite Element Method”)
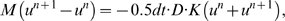
(2)where *M* represents the mass matrix, *K* the stiffness matrix, *dt* the discrete interval between each time step, *u^n^* and *u^n^* ^+1^ the concentration of fluorescent molecules at the *n*th and *(n+1)*th time step, respectively. Here the matrices were assembled using the finite element method to incorporate the geometry of the cell. Zero flux was assumed at cell boundary.

For a given initial fluorescent concentration *u^n^* and an assigned diffusion coefficient, the fluorescent concentration at the next time step, *u^n^* ^+1^, can be computed based on a simple transformation of Eq. (2):
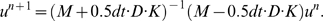



With the interval between each time step *dt* set to be 0.0313 sec, numerical convergence of the FE method was confirmed by comparing the estimated diffusion coefficients and simulated diffusion results with those on a higher resolution mesh and a smaller time step.

According to Eq. (2), there is a linear relationship between the weighted change of concentration in time (WCCT), *M* (*u^n^* ^+1^−*u^n^*), and the weighted discrete Laplacian of concentration (WDLC), −0.5*dt*·*K*·(*u^n^*+*u^n^* ^+1^). Therefore, based on the fluorescence concentration at two consecutive time steps, the diffusion coefficient can be estimated by linear fitting between these two quantities using the least square method (Figure 2). The calculated diffusion coefficient is then compared with the originally assigned diffusion coefficient to assess the accuracy of our method. The whole process of computational simulation to assess and verify the accuracy of our FE and diffusion model is illustrated in Figure 3. All the computer-simulated concentration images were processed using a median filter with a window sized at 10×10 pixels (Figure 3).

### Diffusion Analysis and Simulation Based on FRAP Experiments

Similarly, the apparent diffusion coefficients of the Src biosensors in FRAP experiments were obtained by computing the least-square linear fitting between the WDLC and the WCCT of the concentration images. The diffusion coefficients were then used to simulate and predict the fluorescence recovery maps for comparison with the experimental concentration images (Figure 6).

Different from the computer simulation which covers the entire cell, most of the FRAP images were captured with the 100× objective, so only part of the cell was captured in the image in some occasions. Therefore there may be fluxes across the image boundary, which is not part of the cell boundary. In these cases, instead of zero flux boundary conditions (BCs), the BCs were computed with the apparent diffusion coefficient during linear fitting, by estimating both parameter *D* and *r_0_* in Eq. S7 [46]. Using this linear regression procedure, one estimated apparent diffusion coefficient can be computed with every pair of concentration maps (FRET ratio) *u^n^* and *u^n+1^*. The apparent diffusion coefficient was obtained by averaging the estimated diffusion coefficients of several time intervals. This strategy bears some similarity with the classic FRAP analysis where one apparent diffusion coefficient is obtained by fitting the complete recovery curve. In addition, it is required that we convert the experimental fluorescent intensity images to concentration maps, and reduce noise by smoothing the images at several stages, as described in details in Text S1, “Pre-processing of FRAP Experimental Images”.

### Error Analysis

Two kinds of error analysis were used to evaluate the accuracy of our diffusion model at each time step. First, the absolute value of the error, *abs(u^n^−est_u^n^)*, was used to show the difference between the simulated concentration map with experimental images. Here *est_u^n^* and *u^n^* denote the simulated and experimental concentration maps at the *n*th time step, respectively.

The accuracy of our diffusion model was further evaluated by computing the coefficient of determination, which measures the percentile of total variation in the data that can be explained by the mathematical model [75]. In our diffusion model, the coefficient of determination, *R^2^*, is equivalent to the square of the linear correlation coefficient between WCCT {*x_i_*} and WDLC {*y_i_*}. The linear correlation coefficient between these two data sets {*x_i_*} and {*y_i_*} is defined as
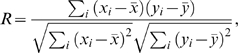
where *x̅* and *y̅* are the mean values of {*x_i_*} and {*y_i_*} respectively. To smooth the data and reduce the computational noise, the data set of WDLC {*y_i_*} and WCCT {*x_i_*} was divided into ten equal intervals along the x-axis and averaged at each interval before computing the coefficient of determination.

### Statistical Analysis

For statistical analysis of the estimated apparent diffusion coefficients and the coefficients of determination, we used the Bonferroni multiple comparison test of means at 95% confidence interval, which is provided by the *multcompare* function in the MATLAB statistics toolbox (The MathWorks, Natick, MA). The estimated apparent diffusion coefficients were selected based on the criteria described in Text S1, “Including Estimated Coefficients in Statistical Analysis”.

### Subtracting Diffusion

The FRET ratio images (CFP intensity/ YFP intensity) were used to quantify the Src activity, or the concentration of phosphorylated Src biosensor. As shown in Figure 1, the FRET signals originated from the diffusion of the biosensor at any given time (Figure 1C) was simulated by using the FRET image of the previous time step (Figure 1A) and the apparent diffusion coefficient estimated by previous FRAP experiments of the biosensor. This simulated FRET image (Figure 1C) was then subtracted from the recorded apparent FRET image at the given time (Figure 1B) to obtain the transient FRET changes, which represents the actions of Src kinase activity on the biosensor between these two time steps (Figure 1D). These transient FRET changes were then iteratively added to the initial FRET image obtained right after EGF application to reconstruct the diffusion-corrected FRET images, which represents the cumulative Src kinase activity on its relatively immobile substrate molecules, such as those in the focal adhesion complex.

## Supporting Information

Text S1. Supplementary Methods.
(0.09 MB DOC)

Figure S1. Triangular mesh. The triangular mesh used in FE analysis. Panel (A) shows the complete mesh. Panel (B) shows a close-up view of the rectangular region as indicated in (A).
(1.62 MB TIF)

Figure S2. Biosensor structure. The structure of Src biosensor and its activation mechanism. Left panel: When the Src biosensor is inactive, the energy transfer in the biosensor with a non-phosphorylated substrate is strong due to the close proximity of YFP to CFP. Right panel: Active Src causes the phosphorylation of the substrate peptide that binds to the SH2 domain in the biosensor. This event induces a conformational change that pulls YFP away from CFP, decreases the energy transfer, and increase the FRET ratio defined by CFP/YFP intensity.
(0.20 MB TIF)

Movie S1. Photobleaching Lyn-Src Biosensor in a HeLa cell. The photobleaching and recovery of a HeLa cell expressing Lyn-Src biosensor.
(7.29 MB MOV)

Movie S2. Diffusion corrected Src activity in lipid rafts. The apparent FRET signals of the Lyn-Src biosensor upon EGF stimulation is compared with the Src activity maps reconstructed by subtracting the diffusion of Lyn-Src biosensor. Upper panel (apparent FRET signals): displays no significant spatial gradient in Src activation. Lower panel (after subtracting diffusion): shows high Src activity at discrete spots near cell periphery upon EGF stimulation.
(4.81 MB MOV)
