# Supplementary material for: The Spatiotemporal Pattern of Src Activation at Lipid Rafts Revealed by Diffusion-Corrected FRET Imaging
Source: PLoS Comput Biol. 2008 Jul 25;4(7):e1000127. doi: 10.1371/journal.pcbi.1000127 (PMC2517613; doi:10.1371/journal.pcbi.1000127)
Supplement: Text S1. — Supplementary Methods. (0.09 MB DOC) [file pcbi.1000127.s001.doc]

# Supplementary Materials

**Supplementary Methods**

**The Formulation of the Finite Element Method**

The finite element method has been used to solve partial differential equations since 1960 (1). The most attractive feature of this method is the ability to handle complex geometric features in both two-dimensional and three-dimensional space with relative ease, because the discretization process is based on an abstract geometric domain $\Omega$ (2). The process of finite element discretization for the diffusion equation with boundary conditions is presented in details here (3). The mathematical proof of the equivalence between the diffusion equation with BCs (see Eq. (S1)-(S2)) and the weak form (see Eq. (S4)), and the approximation property of the Galerkin discretization was described by Brenner et al.(4).

The diffusion equation with boundary conditions (BC’s) is given by

$$\left\{\begin{matrix} \frac{\partial u(x,y,t)}{\partial t}=D\cdot\Delta u(x,y,t),\text{ in a cell; (S1)} \\ \frac{\partial u(x,y,t)}{\partial n}=r(x,y,t),\text{ at the cell edge. (S2)} \end{matrix}\right.$$

According to the Crank-Nicholson Scheme, equation (S1) can be approximated by

(S3)

Let $dt=t_{n+1}-t_{n}$be the time stepping size, multiplying both sides of Eq. (S3) by an arbitrary test function$v$and integrating in the domain Ω, we have the equation in its weak form

(S4)

Moving$dt$to the right hand side, integrating by parts and applying the boundary condition in equation (S2), we have

(S5)

In the finite element method, a triangular mesh is built within the cell edge for the discretization of the weak form (Figure S1). Approximated by the Galerkin discretization, we solve for $u_{h}^{n+1}$in the space $V=span\{\varphi_{1},\cdots,\varphi_{m}\}$, such that

, (S6)

for every $\varphi_{i}$ which is a piecewise linear function on the triangular mesh that has the value 1 on the *ith* node of the mesh and the value 0 on all the other nodes. Because $u_{h}^{n}=\sum_{j=1}^{m} u_{j}^{n}\varphi_{j}$ and $r_{h}^{n}=\sum_{j=1}^{m} r_{j}^{n}\varphi_{j}$, Eq. (S6) can be expanded to:

;

and converted to the matrix form with some rearrangement of terms:

, (S7)

where the mass matrix $M=[m_{ij}]=\left[\int_{\Omega} \varphi_{i}\varphi_{j}dx\right]$, the stiffness matrix $K=[k_{ij}]=\left[\int_{\Omega} \nabla\varphi_{i}\nabla\varphi_{j}dx\right]$, the solution vector $u=[u_{i}^{n}]$, and the vector of BC’s . The values of vector *r_0_* corresponds to the BC’s at the cell edge, and it is zero at internal boundaries (see “Sample MATLAB Scripts”, function estimate_diffusion_coefficient). When the boundary flux is zero, Eq. (S7) can be simplified,

(S8)

In the FE method, the mesh size is an important factor that may affect the accuracy of image analysis and modeling. We have evaluated the effect of mesh size on the estimated apparent diffusion coefficient and the FRET images after diffusion subtraction. The results with varying mesh sizes (0.05-0.25 μm) were compared and confirmed to show no significant difference. Therefore, mesh sizes within the examined range should not have significant impact on the computational results.

**Pre-processing of FRAP Experimental Images**

All the images from FRAP experiments were background-subtracted and then processed using a modified adaptive filter with a window sized at $40\times40$ pixels. This modified adaptive filter was constructed based on the low-pass adaptive filter wiener2 in the MATLAB image processing toolbox (The MathWorks) (5), which smoothes the noise adaptively based on the local variation of the image in the filter window. The boundary of the reference cell image was determined based on the fluorescence intensity, and used throughout the analysis, given the fact that the cells did not change locations and geometry significantly during the FRAP procedure. The filtered pre-bleach images were further smoothed with a temporal median filter to produce a reference image. The post-bleach images were normalized by the reference image to convert the fluorescence images into concentration maps to eliminate the effect of geometric factors, such as depth of the cytoplasm and membrane folding. A second adaptive filter was further used to smooth out the normalized images. An additional median filter of size 3 was used in the temporal space where necessary. Linear fitting between the WDLC and the WCCT of these processed concentration images was used to calculate the apparent diffusion coefficients of different Src biosensors, which were then applied to simulate and predict the fluorescence recovery images to compare with the experimental data (Figure 6).

**Including Estimated Diffusion Coefficients in Statistical Analysis**

The relative error, *norm(u^n^ - est_u^n^)/norm(u^n^)*, was used to quantitatively compare the estimated concentration map with experimental results, and to decide whether to accept or discard the estimated apparent diffusion coefficient and the coefficient of determination. Among the four groups of cells, 19 cells and 102 diffusion coefficients were included in statistical analysis. The coefficients of determination were discarded if the R values were negative and hence not physically relevant. Altogether 92 coefficients of determination were included in the statistics.

**Model Assumptions**

In the model for photobleaching images, we assumed a uniform diffusion coefficient in a two-dimensional space for different biosensors, with nonzero flux at the cell edge. All the initial conditions and time-space parameters were based on the experimental data. FE analysis and linear regression were applied to estimate the apparent diffusion coefficients of different biosensors. This model appears to fit the best with the motion of the Lyn-Src biosensor (Figures 10 and 11B), but not well with that of the cytosolic-Src biosensor.

**Sample MATLAB Scripts**

A software named ‘fluocell’ has been built to implement our FE-based diffusion analysis procedure. It can be obtained by writing to the corresponding author of this paper. Here we present some sample MATLAB scripts implementing the finite element discretization and linear regression.

% Create the mesh, assemble the mass matrices and the stiffness matrices

[p_image,edge,tri]=create_mesh(smaller_boundary,num_refine);

p = scale_by_magnification(p_image,mag);

[K, M] = assemble_matrices(p,tri);

is_boundary = mark_mesh_boundary(p_image, edge);

% convert concentration to solution vector

num_nodes = size(p_image,2)

u = zeros(num_nodes,num_steps);

est_u = zeros(num_nodes,num_steps);

r = zeros(num_nodes, num_steps);

for i = 1:num_steps,

u(:,i) = concentration_to_vector(med_con(:,:,:,i),p_image);

end;

% Estimate diffusion constant and provide output.

display(sprintf('%20s%20s%20s%20s',...

'diffusion coef', 'residual_1', 'R value'));

dt = dt*num_images_per_layer;

diff_const = zeros(num_steps-1,1);

for i= 1:num_steps-1,

Mdu = M*(u(:,i+1)-u(:,i));

dtKu = -dt*K*0.5*(u(:,i+1)+u(:,i));

[diff_const(i) , est_u(:,i+1) , r(:,i+1), R] = ...

estimate_diffusion_coefficient(u(:,i),u(:,i+1),...

M,K,dt,is_boundary);

relative_residual_norm = norm(u(:,i+1)-est_u(:,i+1),2)/...

norm(u(:,i+1),2);

display(sprintf('%20f%20f%20f%20f', diff_const(i), ...

relative_residual_norm, R));

end;

% Estimate the diffusion coefficient by linear fitting

% Boundary flux is not zero at the cell edge

% Compute the predicted concentration map based on the

% linear diffusion model.

function [diff_const, est_u2, r, R12] = ...

estimate_diffusion_coefficient(u1,u2,M,K,dt, is_boundary);

alpha = 0.5;

beta = 0.5;

Mdu = M*(u2-u1);

dtKu = -dt*K*(alpha*u1+beta*u2);

one_over_D = Mdu\dtKu;

diff_const = 1.0/one_over_D;

r = Mdu-diff_const*dtKu;

r0 = r.*is_boundary;

est_u2 = (M+beta*dt*diff_const*K)\...

((M-alpha*dt*diff_const*K)*u1+r1);

[x,y] = average_data(Mdu, dtKu);

[R,P]=corrcoef(x,y);

R12 = R(1,2);

r = (r-r0)/(diff_const*dt);

return;

**Supplementary References**

1. Clough, R. W. The finite element method in plane stress analysis; 1960; Pittsburgh, Pa.

2. Zienkiewicz, O. C., R. L. Taylor, and J. Z. Zhu. 2005. The Finite Element Method: Its Basis and Fundamentals. Oxford: Elsevier Butterworth-Heinemann.

3. Tai, K., S. D. Bond, H. R. MacMillan, N. A. Baker, M. J. Holst, and J. A. McCammon. 2003. Finite element simulations of acetylcholine diffusion in neuromuscular junctions. Biophys J. 84:2234-2241.

4. Brenner, S. C. and L. R. Scot. 2002. The Mathematical Theory of Finite Element Methods. J. E. Marsden, L. Sirovich, M. Golubitsky, and S. S. Antman, editors. New York: Springer-Verlag.

5. Lim, J. S. 1990. Two-Dimensional Signal and Image Processing. Englewood Cliffs, NJ: Prentice Hall. p. 548, equations 549.544 -- 549.546 p.

# 
