# Supplementary figures and images for: The Spatiotemporal Pattern of Src Activation at Lipid Rafts Revealed by Diffusion-Corrected FRET Imaging
Source: PLoS Comput Biol. 2008 Jul 25;4(7):e1000127. doi: 10.1371/journal.pcbi.1000127 (PMC2517613; doi:10.1371/journal.pcbi.1000127)

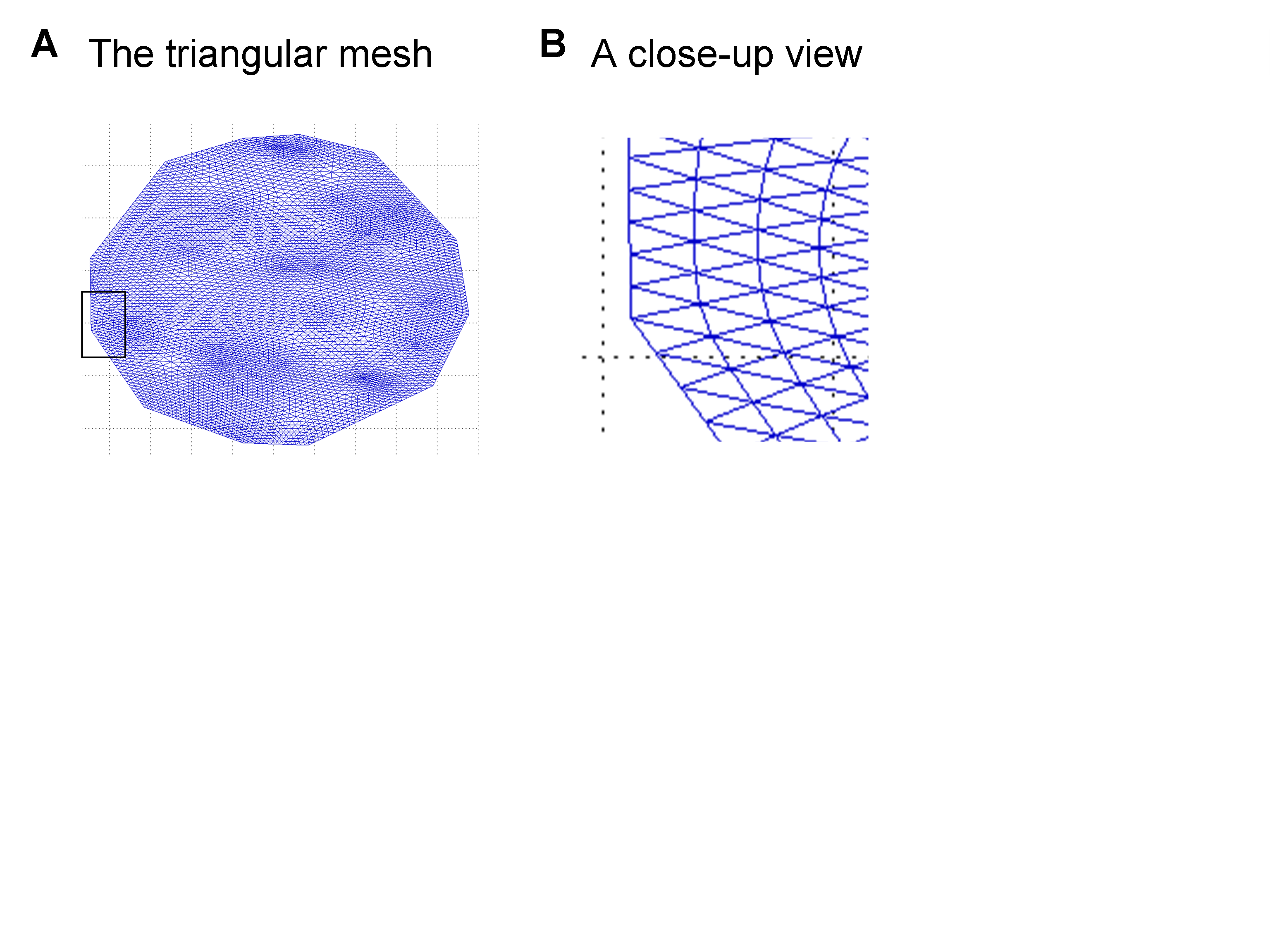

Supplement: Figure S1. — Triangular mesh. The triangular mesh used in FE analysis. Panel (A) shows the complete mesh. Panel (B) shows a close-up view of the rectangular region as indicated in (A). (1.62 MB TIF) [file pcbi.1000127.s002.tif]

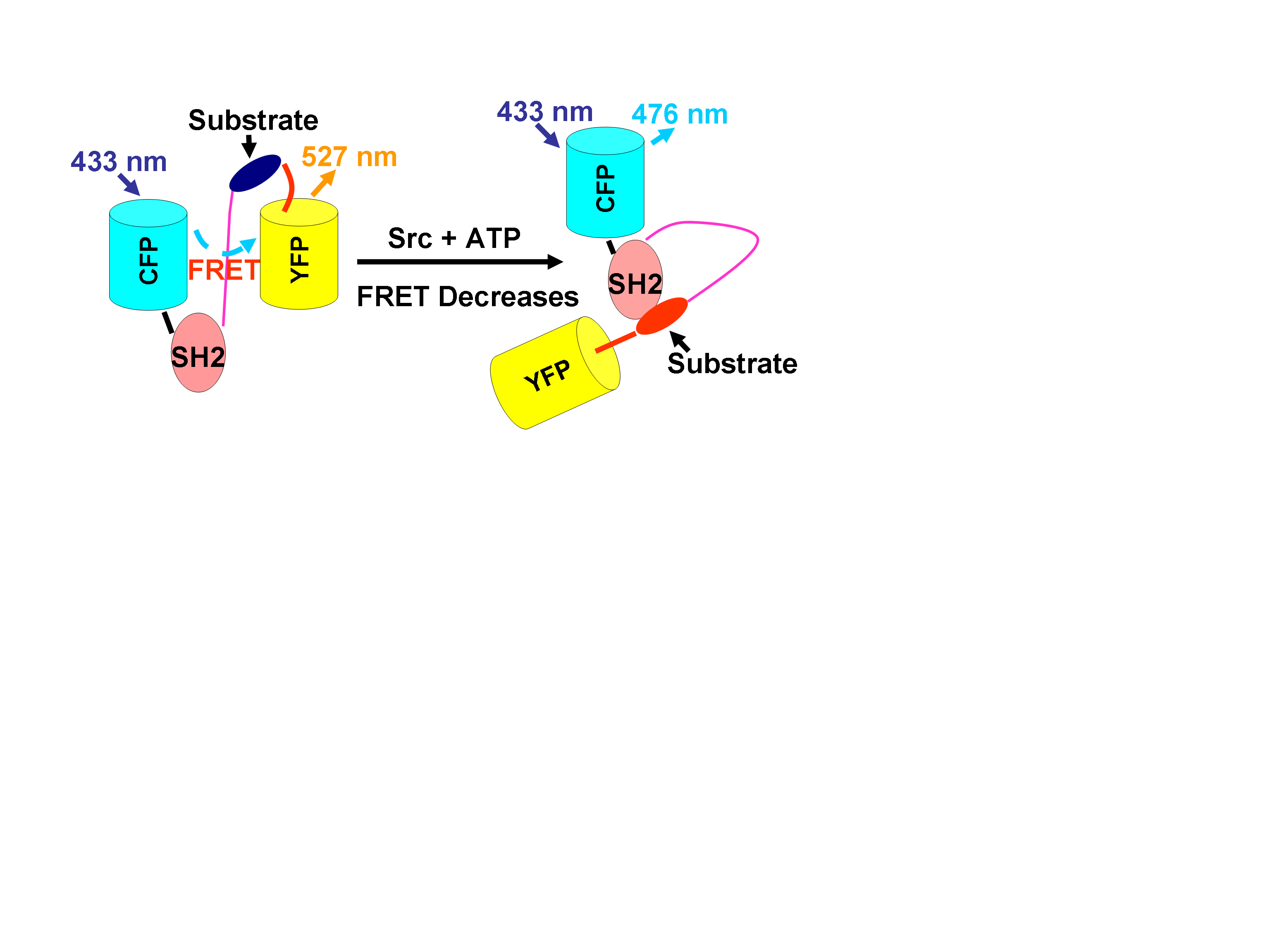

Supplement: Figure S2. — Biosensor structure. The structure of Src biosensor and its activation mechanism. Left panel: When the Src biosensor is inactive, the energy transfer in the biosensor with a non-phosphorylated substrate is strong due to the close proximity of YFP to CFP. Right panel: Active Src causes the phosphorylation of the substrate peptide that binds to the SH2 domain in the biosensor. This event induces a conformational change that pulls YFP away from CFP, decreases the energy transfer, and increase the FRET ratio defined by CFP/YFP intensity. (0.20 MB TIF) [file pcbi.1000127.s003.tif]
